# Genome Sequence of a Canine Parvovirus Strain, CPV-s5, Prevalent in Southern China

**DOI:** 10.1128/genomeA.01141-13

**Published:** 2014-01-09

**Authors:** Yiping Zhu, Yongliang Huang, Yifei Wang, Keren Chen, Xuefeng Niu, Yongwen Luo, Xiaofeng Guo

**Affiliations:** College of Veterinary Medicine, South China Agricultural University, Guangzhou, Guangdong, China

## Abstract

A prevalent new field canine parvovirus type-2a (CPV-2a) strain, CPV-s5, was isolated from the feces of a dog with diarrhea in Shenzhen, China. The genome of CPV-s5 was determined and analyzed, which will facilitate further study of the molecular epidemiology and genetic diversity of CPV-2 field isolates in southern China.

## GENOME ANNOUNCEMENT

Since its sudden emergence in the early 1970s, canine parvovirus type-2 (CPV-2) has been evolving into novel genetic and antigenic variants (CPV-2a, -2b, and -2c) that are distributed throughout the world (1–4). In recent years, infections of CPV-2 in dogs are increasingly frequent in southern China, and the dominant type of CPV is CPV-2a. A virulent field CPV-2 strain, CPV-s5, was isolated from a fecal sample of a puppy with severe diarrhea in Shenzhen, China, in July 2010. The genome sequence of CPV-s5 was determined and analyzed as part of an effort to monitor the genetic diversity of CPV-2 in southern China.

The genome, excluding a partial inverted terminal repeat (ITR) sequence (approximately 300 nucleotides [nt], respectively) was amplified by PCR utilizing *Pfu* DNA polymerase with four pairs of primers. The PCR products were gel purified by the QIAquick gel extraction kit (Qiagen) and sequenced on a 3730 DNA analyzer (Applied Biosystems). The genome of CPV-5s comprises 4,702 nucleotides containing two open reading frames (ORFs). ORF1 (nt 154 to 2160) encodes two nonstructural proteins (NS1 and NS2), and ORF2 (nt 2167 to 4422) encodes two structural proteins (VP1 and VP2).

Whole-genome phylogeny using nucleotide sequences of CPV-2 retrieved from GenBank revealed that CPV-s5 clusters most closely with CPV strains SCO2/2011 and CPV-LZ1, which were isolated from China in 2011 (5). CPV-s5 shares 99.2 to 99.8% nucleotide identity with the CPV-2 isolates from China and 98.8% homology with the feline panleukopenia virus (FPV) strain 193/70. There are remarkable insertion regions in CPV-s5 (nt 4456 to 4515) and SCO2/2011 (nt 4453 to 4512) in the 3′-untranslated region (3′-UTR) compared to other sequenced CPV-2a strains isolated from China. An interesting repeated sequence (nt 4483 to 4515), which appeared previously in nt 4423 to 4459, was found in the insertion.

According to the amino acid variation of Ser297Ala in the VP2 capsid protein, CPV-s5 was classified as a new CPV-2a strain. A rare nucleotide variation at position 1867 (G→A), resulting in the amino acid substitution Glu572Lys in NS1, appeared in strains CPV-5s, SCO2/2011, CPV-LZ1, B-2004, and CPV-339. A specific Ser192Phe mutation was observed only in VP2 of CPV-5s and SCO2/2011. Two VP2 mutations, Tyr324Ile and Thr440Ala, were present in most of the CPV-2a strains isolated from China but not the vaccine strains or strains from other countries.

The present study demonstrates that the CPV-2a strains circulating in southern China have been undergoing some unique variations. Antigenic variations between ﬁeld and vaccine CPV-2 strains may be one of the important reasons leading to immune failure in China. Comprehensive epidemiological and pathogenetic investigations of the new antigenic strains of CPV-2 are needed for effective control of CPV infection in China.

### Nucleotide sequence accession number.

The genome sequence of new CPV-2a strain CPV-s5 has been deposited in GenBank under the accession no. KF638400.
